# Draft Genome Sequence of *Exophiala* Strain HKRS030, a Fungus Capable of Reducing Iron but Not Nitrate

**DOI:** 10.1128/mra.00615-22

**Published:** 2023-02-13

**Authors:** Zachary T. Burton, Harry D. Kurtz

**Affiliations:** a Department of Biological Sciences, Clemson University, Clemson, South Carolina, USA; University of California, Riverside

## Abstract

*Exophiala* strain HKRS030 was isolated from a cryptoendolithic community found in the Grand Staircase-Escalante National Monument (GSENM) in southern Utah, USA. *Exophiala* strain HKRS030 was observed to dissimilatorily reduce iron(III) while being unable to reduce nitrate.

## ANNOUNCEMENT

While enriching for acidophilic iron-reducing bacteria using modified medium 2, containing solution 1 and SL8 (R2A medium) medium (pH 3) supplemented with 0.1% glucose and 250 μM FeCl_3_ (1), we isolated a fungus from a cryptoendolithic community from a crushed sample of Navajo sandstone from the Grand Staircase-Escalante National Monument (GSENM) in southern Utah, USA, which was identified to be a member of the fungal genus *Exophiala* by small-subunit (SSU) ribosomal DNA (rDNA) sequence analysis and was designated strain HKRS030. The host community was from the Navajo sandstone formation near the Cosmic Ashtray in the GSENM (37°41′06.69″N, 111°19′11.64″W) (sample HW07-04) (2). Using the ferrozine assay (3), HKRS030 was shown to reduce Fe^3+^ to Fe^2+^ in a dissimilatory fashion. Other fungi capable of iron reduction also reduce nitrate to nitrite (4). HKRS030 was tested for the ability to reduce nitrate using the nitrite assay (5) and was found to lack the ability to reduce nitrate. Due to this unique physiology, a draft genome sequence was obtained.

DNA was isolated from HKRS030 grown in R2A broth (pH 3) supplemented with 0.9% glucose and 250 μM FeCl_3_ by using the DNeasy UltraClean microbial kit (Qiagen, Hilden, Germany). DNA concentrations were determined using a Qubit fluorometer (2.4 ng/mL), and then DNA was prepared for sequencing using the Illumina Nextera Flex library preparation kit and sequenced using the NovaSeq platform (Illumina, San Diego, CA) by the Clemson University Genomics and Bioinformatics Facility (CUGBF). Sequence data were quality checked using FastQC (v 0.11.9) (6), trimmed using TrimGalore (v 0.6.7) (7), and quality checked after trimming using FastQC. A *de novo* assembly was produced using SPAdes (v 3.15.4) (8), to obtain a preliminary genome annotation using DFAST (v 1.2.6) (9). Using this annotation, rRNA gene sequences were identified and used to search GenBank via BLAST (10) for strain identification. The genome was reassembled using the Exophiala oligosperma reference genome (GenBank accession number JYCA00000000) from GenBank with SSPACE (v 2.1) (11). BUSCO (v 5.3.2) (12) (with fungal database Fungi Odb10) and Anvi’o (v 7.0) (13) (with default settings) were used to determine the completeness of the genome. Using the programs BWA (v 0.7.17) and SAMtools (v 1.15.1), the average coverage of the genome was determined using a script acquired from Rooksana Noorai (14). The SAMtools mpileup program (15) was used to determine the coverage per contig. RaGOO (v 1.0) (16) was used to bin the contigs into scaffolds, using the E. oligosperma genome as a reference.

Postprocessing, the assembly had 95 scaffolds, with a scaffold *N*_50_ value of 5.39 Mb and a contig length 0.93 Mb, averaging 3 contigs per scaffold. Anvi’o and BUSCO were used to gauge genome completeness and agreed that the genome was 98.5% complete and was 60.19 Mb, with a GC content of 49.9%. The average nucleotide identity (ANI) for HKRS030 was calculated to be 99.98% with respect to E. oligosperma (GenBank accession number JYCA00000000) using a web-based two-way ANI calculator (http://enve-omics.ce.gatech.edu/ani/) (17). However, gaps in the assembly need to be filled to bring our assembly into agreement with the number of contigs, i.e., 11 to 13 contigs, observed for other members of the *Exophiala* genus with completed genome sequences (18).

### Data availability.

This whole-genome shotgun project has the genome accession number JAHWDD000000000, with BioProject accession number PRJNA744509. This version of the project (version 1) has the accession number JAHWDD010000000 and consists of sequences JAHWDD010000001 to JAHWDD010000093.

## References

[1] Biebl H, Pfennig N. 1978. Growth yields of green sulfur bacteria in mixed cultures with sulfur and sulfate reducing bacteria. Arch Microbiol 117:9–16. doi:10.1007/BF00689344.

[2] Kaur S, Kurtz HD, Jr. 2019. Core bacterial community composition of a cryptoendolithic ecosystem in the Grand Staircase-Escalante National Monument, Utah, USA. Microbiologyopen 8:e00707. doi:10.1002/mbo3.707. 30079546 PMC6528646

[3] Hammes E, Floyd M, Kurtz HD, Jr. 2013. An iron(II) binding EPS and an assessment of microbial diversity in association with the EPS: implications for iron cycling in the Jurassic Navajo Sandstone. J Arid Environ 97:49–55. doi:10.1016/j.jaridenv.2013.05.006.

[4] Ottow JCG, von Klopotek A. 1969. Enzymatic reduction of iron oxide by fungi. Appl Microbiol 18:41–43. doi:10.1128/am.18.1.41-43.1969. 16349855 PMC377882

[5] Taylor HB, Kurtz HD, Jr. 2020. Composition, diversity, and activity of aerobic ammonia-oxidizing bacteria and archaea in the intertidal sands of a Grand Strand South Carolina beach. Microbiologyopen 9:e1011. doi:10.1002/mbo3.1011. 32126588 PMC7221436

[6] Andrews S. 2010. FastQC: a quality control tool for high throughput sequence data. https://www.bioinformatics.babraham.ac.uk/projects/fastqc.

[7] Krueger F. 2015. Trim Galore. https://www.bioinformatics.babraham.ac.uk/projects/trim_galore.

[8] Prjibelski A, Antipov D, Meleshko D, Lapidus A, Korobeynikov A. 2020. Using SPAdes de novo assembler. Curr Protoc Bioinformatics 70:e102. doi:10.1002/cpbi.102. 32559359

[9] Tanizawa Y, Fujisawa T, Nakamura Y. 2018. DFAST: a flexible prokaryotic genome annotation pipeline for faster genome publication. Bioinformatics 34:1037–1039. doi:10.1093/bioinformatics/btx713. 29106469 PMC5860143

[10] Altschul SF, Gish W, Miller W, Myers EW, Lipman DJ. 1990. Basic local alignment search tool. J Mol Biol 215:403–410. doi:10.1016/S0022-2836(05)80360-2. 2231712

[11] Boetzer M, Henkel CV, Jansen HJ, Butler D, Pirovano W. 2011. Scaffolding pre-assembled contigs using SSPACE. Bioinformatics 27:578–579. doi:10.1093/bioinformatics/btq683. 21149342

[12] Simão FA, Waterhouse RM, Ioannidis P, Kriventseva EV, Zdobnov EM. 2015. BUSCO: assessing genome assembly and annotation completeness with single-copy orthologs. Bioinformatics 31:3210–3212. doi:10.1093/bioinformatics/btv351. 26059717

[13] Eren AM, Esen OC, Quince C, Vineis JH, Morrison HG, Sogin ML, Delmont TO. 2015. Anvi’o: an advanced analysis and visualization platform for ’omics data. PeerJ 3:e1319. doi:10.7717/peerj.1319. 26500826 PMC4614810

[14] Noorai RE, Freese NH, Wright LM, Chapman SC, Clark LA. 2012. Genome-wide association mapping and identification of candidate genes for the rumpless and ear-tufted traits of the *Araucana* chicken. PLoS One 7:e40974. doi:10.1371/journal.pone.0040974. 22844420 PMC3402462

[15] Li H, Handsaker B, Wysoker A, Fennell T, Ruan J, Homer N, Marth G, Abecasis G, Durbin R, 1000 Genome Project Data Processing Subgroup. 2009. The Sequence Alignment/Map format and SAMtools. Bioinformatics 25:2078–2079. doi:10.1093/bioinformatics/btp352. 19505943 PMC2723002

[16] Alonge M, Soyk S, Ramakrishnan S, Wang X, Goodwin S, Sedlazeck FJ, Lippman ZB, Schatz MC. 2019. RaGOO: fast and accurate reference-guided scaffolding of draft genomes. Genome Biol 20:224. doi:10.1186/s13059-019-1829-6. 31661016 PMC6816165

[17] Rodriguez-R LM, Konstantinidis KT. 2016. The enveomics collection: a toolbox for specialized analyses of microbial genomes and metagenomes. PeerJ Preprints 4:e1900v1. doi:10.7287/peerj.preprints.1900v1.

[18] Burton Z. 2021. Investigation of dissimilatory iron reduction by Exophiala HKRS030. M.Sc. thesis. Clemson University, Clemson, SC.

