# Correction for Burton and Kurtz, “Draft Genome Sequence of *Exophiala* Strain HKRS030, a Fungus Capable of Reducing Iron but Not Nitrate”

**DOI:** 10.1128/mra.00223-25

**Published:** 2025-04-16

**Authors:** Zachary T. Burton, Harry D. Kurtz

## ERRATUM

Volume 12, no. 3, e00615-22, 2023. https://doi.org/10.1128/mra.00615-22 Page 2, lines 3-4 of Acknowledgments should read “Institute of General Medical Sciences of the National Institutes of Health (grants P20GM109094 and P20GM139769).”

